# Using meta-regression analyses in addition to conventional systematic review methods to examine the variation in cost-effectiveness results – a case study

**DOI:** 10.1186/s12913-015-1230-4

**Published:** 2016-01-20

**Authors:** Laura T. Burgers, Fleur T. van de Wetering, Johan L. Severens, W. Ken Redekop

**Affiliations:** 1 Institute for Medical Technology Assessment, Erasmus University Rotterdam, Rotterdam, The Netherlands; 2 Institute of Health Policy & Management, Erasmus University Rotterdam, Rotterdam, The Netherlands; 3 Cochrane Netherlands, Julius Center for Health Sciences and Primary Care, University Medical Center Utrecht, Utrecht, The Netherlands

**Keywords:** Systematic review, Cost-effectiveness, Stents, Modelling, Meta-regression

## Abstract

**Background:**

Systematic reviews of cost-effectiveness analyses summarize results and describe study characteristics. Variability in the study results is often explained qualitatively or based on sensitivity analyses of individual studies. However, variability due to input parameters and study characteristics (e.g., funding or study quality) is often not statistically explained. As a case study, a systematic review on the cost-effectiveness of drug-eluting stents (DES) versus bare-metal stents (BMS) using meta-regression analyses is performed to explore the usefulness of such methods compared with conventional review methods.

**Methods:**

We attempted to identify and review all modelling studies published until January 2012 that compared costs and consequences of DES versus BMS. We extracted general study information (e.g., funding), modelling methods, values of input parameters, and quality of the model using the Philips et al. checklist. Associations between study characteristics and the incremental costs and effectiveness of individual analyses were explored using regression analyses corrected for study ID.

**Results:**

Sixteen eligible studies were identified, with a combined total of 508 analyses. The overall quality of the models was moderate (59 % ± 15 %). This study showed associations (e.g., type of lesion) that were expected (based on individual studies), however the meta-regression analyses revealed also unpredicted associations: e.g., model quality was negatively associated with repeat revascularizations avoided.

**Conclusions:**

Meta-regressions can be of added value, identifying significant associations that could not be identified using conventional review methods or by sensitivity analyses of individual studies. Furthermore, this study underlines the need to examine input parameters and perform a quality check of studies when interpreting the results.

**Electronic supplementary material:**

The online version of this article (doi:10.1186/s12913-015-1230-4) contains supplementary material, which is available to authorized users.

## Background

Economic evaluations are increasingly used to assist in decision making of interventions. Often for a specific decision problem different economic evaluations are conducted. The results of these studies may differ substantially between studies: from interventions being dominated to being dominant. Therefore, it is necessary that systematic reviews are performed to summarize the results of the individual economic evaluations. Besides summarizing the study characteristics and results it would be interesting to explain statistically the variability in the incremental costs and incremental effects and thus the conclusions. Differences can exist due to differences in values used for input parameters, perspective, time horizon and other factors. Some differences could easily be explained by the values that were used for the input parameters, since for some input parameters a linear relationship with the outcomes exists. For example, an increase in initial intervention costs will lead to an increase in the incremental costs, ceteris paribus. Often these variations are explained by sensitivity analyses of individual studies. Other associations with input parameters that do not have a linear association with the outcome (e.g., probabilities leading to changes in costs and effects) or study characteristics (e.g., funding) could be identified using meta-regression analyses in addition to conventional systematic review methods. Meta-regression analyses are currently used to combine the results of clinical trials and to investigate the effect of methodological diversity of the studies on the results [1]. To explain the variability in the incremental costs and incremental effects of cost-effectiveness analysis (CEA) it could be useful to apply these meta-regression analyses in systematic reviews of economic evaluations.

The aim of this study is to explore the usefulness of meta-regression analyses in systematically explaining the variability in the results compared with conventional review methods and sensitivity analyses of individual studies. Meta-regression analyses may be useful if they provide more information, in terms of associations with the outcomes, than conventional systematic reviews and sensitivity analyses. Many economic evaluations have estimated the cost-effectiveness of drug-eluting stents (DES) versus bare-metal stents (BMS) for the treatment of patients with coronary artery disease. The results between the studies vary considerably, which makes this decision problem a good case study to explore if meta-regression analyses are of added value. Systematic reviews [2–4] on the cost-effectiveness of DES versus BMS have been performed but did not explore statistically the causes of the variability in incremental costs and incremental effects between the studies. Associations with the incremental outcomes (costs, quality-adjusted life years and repeat revascularizations avoided) will be identified in this study. Besides the ‘known’ factors (e.g., age, type of lesion, price of stents, relative risk repeat revascularisations avoided) explaining the cost-effectiveness of DES versus BMS we will identify associations that could only be identified at a meta-level such as the quality of the studies and funding.

## Methods

### Inclusion and exclusion criteria

A systematic literature search was performed to identify all English-language (online or print) publications (at any time before January 2012) of CEAs using decision analytic models to compare the costs and consequences of DES (sirolimus-eluting stent (SES), paclitaxel-eluting stent (PES), everolimus or zotarolimus-eluting stent (ZES)) versus BMS for patients who require a stent implantation due to an atherosclerotic lesion of the coronary artery. The effectiveness of the studies had to be expressed in quality adjusted life years (QALY) or in disease specific measures such as repeat revascularizations avoided, TLR (target lesion revascularization) and TVR (target vessel revascularization). Furthermore, studies were only included if they reported results in enough detail to enable separation of incremental costs from incremental effects. There was no restriction on the perspective used in the economic evaluation. Reviews, editorials and abstracts were not included in the review.

Studies were identified using electronic databases (PubMed, EMbase, NHS EED, Cochrane Library and INAHTA) and by scanning reference lists of eligible articles. The full search strategies for EMbase and PubMed are presented in Additional file 1. To ensure that all relevant publications were identified in the CRD (NHS EED and HTA) and Cochrane Library databases we limited the search terms to “stent” and “stents”. These terms were searched in “any field” for CRD and in “title, abstract, keywords” for Cochrane Library. We also included the relevant publications found in the reviews by Ligthart et al. [4], Hill et al. [2], and Neyt et al. [3].

### Data extraction

One reviewer (LB) screened the titles and abstracts identified through the searches. The full text evaluation was performed by two reviewers (LB & FW) and discrepancies were discussed and resolved by consensus or by consulting a third reviewer (WR). Various parameters (Tables 1 and 3) were extracted from the relevant publications by one reviewer (LB). The parameters chosen in the regression analyses were the most likely general study characteristics (e.g., population, time horizon, funding) that are reported in conventional systematic reviews. In addition, we added the most important input parameters (e.g., cost of procedure, relative risk of repeat revascularization, probability of repeat revascularization, utilities) that are used in the model to estimate the cost-effectiveness. These key parameters are often varied in deterministic sensitivity analyses. Costs were converted to Euros [5] and corrected for inflation if necessary [6] to present the costs as 2012 Euros. Furthermore, we wanted to see if modelling assumptions (e.g., oculo-stenotic effect) were of influence on the incremental outcomes. All assumptions reported in the studies were monitored. Lastly, two reviewers (LB & FW) independently assessed the quality of the models using the Philips et al. checklist [7] for the assessment of model-based economic analyses. The Philips checklist is a framework based on existing guidelines on the use of decision analytic modelling in health technology assessments. The checklist is structured in three themes: a) structure, which focusses on the scope and mathematical structure; b) data, which examines data identification and uncertainty methods; and c) consistency, which assesses the overall quality of the model based on the publication. Both overall study quality and the quality per theme were given a score from 0-100 %, which was calculated by dividing the sum of the questions answered positively by the total number of relevant questions. Since some questions were not relevant for all studies (e.g., questions concerning quality-of-life values) the denominator could differ between studies.

**Table 1:** Description economic evaluations

Study	Year	Country	# Analyses	Horizon (months)	Model	Funding^b^	Subgroups	Comparison	Price per stent (2012 €)	Price difference DES vs BMS (2012 €)	# Stents per procedure	Quality (%)^a^
Ekman et al. [15]	2004	Sweden	66	12,24	DT	Yes	High risk, diabetes, type of lesion, type of vessel	BMS vs	NS		1.1-1.8	41
								PES	NS	693-1271		
Hill et al. [22]	2004	UK	36	12-60	STM	No	High risk, # vessels	BMS vs	679		1.3,2.4	77
								DES	1607	929		
Tarricone et al. [19]	2004	Italy	10	12	DT	Yes	# vessels, diabetes, type of lesion, type of vessel	BMS vs	NS		1.2 – 2.6	46
								SES	NS	0		
Bowen et al. [21]	2005	Canada	50	12	DT	No	Post MI, diabetes, type of lesion	BMS vs	531		1.23–2.26	61
								DES	1681	1150		
Mittmann et al. [13]	2005	Canada	8	12	DT	NS		BMS vs	522		1.5	50
								SES	2062	1540		
								PES	2062	1540		
Shrive et al. [17]	2005	Canada	11	LT	STM	Yes	Diabetes, age	BMS vs	430		1.05–1.75	56
								SES	1246-3114	816-2685		
Mahieu et al. [12]	2006	Belgium	31	12	DT	NS	Diabetes, type of lesion, type of vessel	BMS vs	NS		1	32
								SES	NS	731-1306		
								PES	NS	731-1306		
Hill et al. [2]	2007	UK	172	12	STM	No	High risk, elective	BMS vs	485		1-2	80
								SES	1700-1774	1215-1289		
								PES	1621-1696	1136-1211		
Kuukasjarvi et al. [23]	2007	Finland	2	24	DT	No		BMS vs	NS		NS	33
								DES	NS	NS		
Neyt et al. [8]	2007	Belgium	59	12	DT	NS	Diabetes, # vessels, type of lesion	BMS vs	553-1106		1.09–1.97	72
								DES	553-1659	0-1106		
Polanczyk et al. [18]	2007	Brazil	4	12, LT	STM	Yes		BMS vs	831-1390		1.2	56
								SES	3169	1779, 2337		
Bischof et al. [14]	2009	USA	4	36	STM	No		BMS vs	NS	NS	NS	76
								SES	NS			
								PES	NS			
Goeree et al.[24]	2009	Canada	45	24	DT	No	Diabetes, type of lesion, type of vessel	BMS vs	470		1.1–2.37	52
								DES	1486	391-1016		
Ferreira et al. [16]	2010	Brazil	1	26	DT	No		BMS vs	1883		NS	36
								PES	5272	3390		
Jahn et al. [10, 11]	2010	Austria	6	84	DES	No	Diabetes, type of lesion	BMS vs	NS		1.24	47
								DES	NS	NS		
Remak et al. [20]	2010	UK	3	48	STM	Yes		BMS vs	433		1.11	62
								ZES	1175	742	1.12-1.4	

^a^ Philips checklist 2006: scale 0-100 %

^b^ Yes: manufacturer; No: funded by government or not funded

*DES* discrete event simulation, *DT* decision tree, *LT* life time, *vs* versus, *MI* myocardial infarction, *NS* not stated, *STM* state-transition model, *# vessels* number of vessels treated

### Analysis

The influence of modelling methods, the choice of parameters and the quality of the models on the main outcomes (incremental costs, incremental QALYs and absolute risk reduction repeat revascularizations) were analysed both quantitatively and qualitatively. Associations between parameters and the outcomes were assessed by identifying outliers found on cost-effectiveness planes. Furthermore, several bivariate linear regressions were estimated to confirm the associations and also to measure the influence of other parameters on the outcomes. Including associations that could be predicted beforehand (e.g., type of lesion, price stent) are included in the regression analyses since it could be seen as a validation check if the analyses also show these associations. Multivariate analyses with all of the parameters that were significant in the bivariate analyses could not be performed due to a high frequency of missing values caused by incomplete reporting.

We included every subgroup or sensitivity analysis found in a study as long as incremental costs or incremental effectiveness were provided or could be calculated. As a result, our meta-regression analyses were based on many more observations than the number of studies that were included. Since Hill et al. [2] provided more than 30 % of the observations used in our study; we incorporated study ID as a random effect in the regression models. Some studies reported both incremental effects and incremental QALYs for a specific analysis. Since the incremental costs associated with both outcomes is the same we only included one of the two analyses for the regression analyses on the incremental costs to avoid double counting. Data management and all statistical analyses were performed with SPSS 19.0 (SPSS Inc., Chicago, IL, USA). The level of measurement was ordinal or ratio, depending on the covariate. The model assumptions and study characteristics (e.g., funding) were measured at an ordinal scale. Input parameters such as the probability of repeat revascularization were measured at a ratio scale. Conclusions about statistical significant were based on an alpha level of 5 %.

## Results

**Fig. 1:**
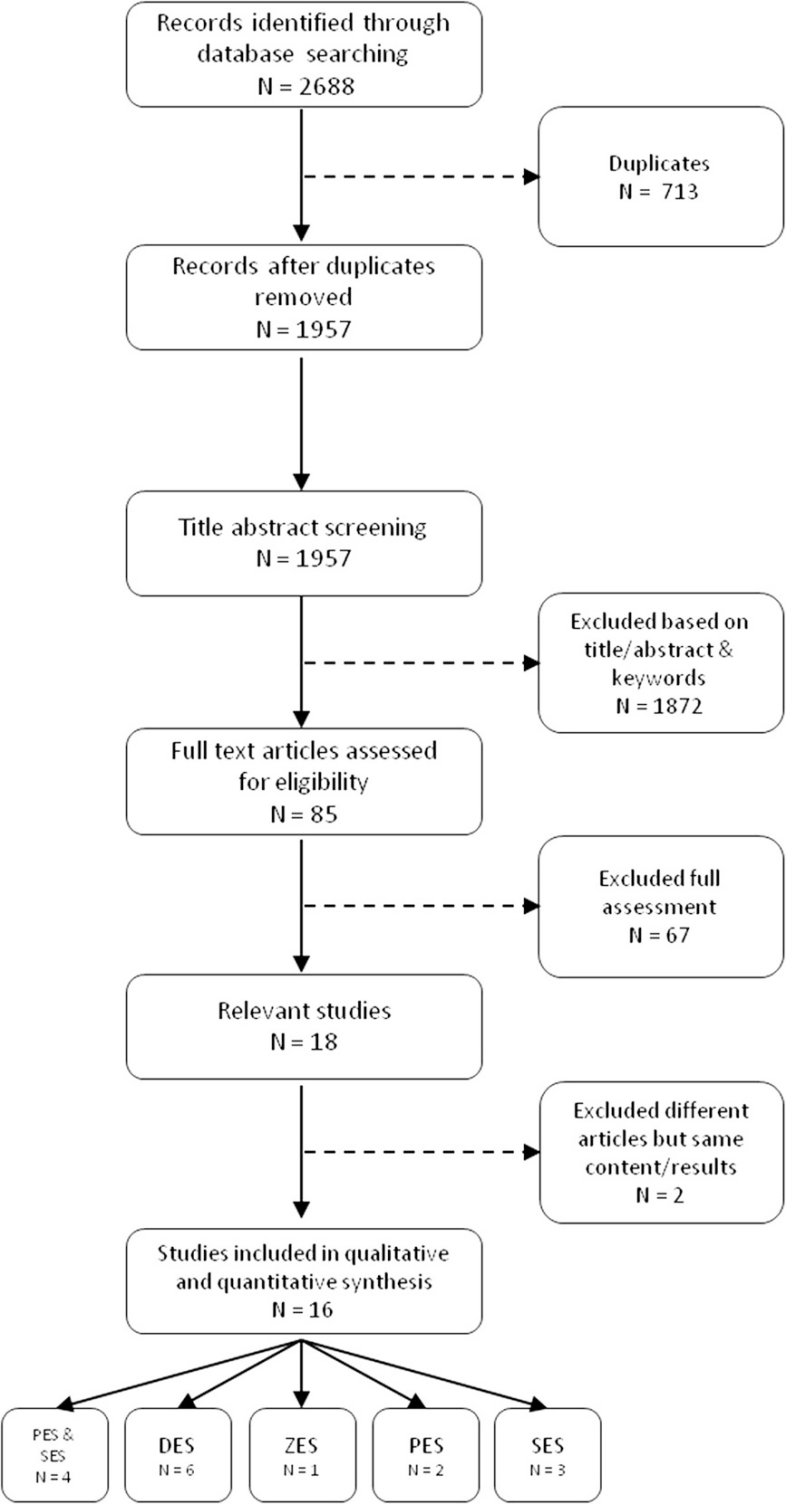
Flow of studies through the review process. PES: paclitaxel eluting stent; SES: sirolimus eluting stent; ZES: zotarolimus eluting stent; DES: drug eluting stent

Figure 1 presents the process of identifying relevant publications in line with PRISMA guidelines (Additional file 2). Of the 1957 potentially relevant publications, 1872 were excluded based on title, abstract and keywords. Full-text evaluation was performed for 85 articles leading to 18 relevant studies. Reasons to exclude studies after a full text assessment were: lack of a model (*n* = 24), no original CEA (*n* = 22), language other than English (*n* = 8), no relevant outcome (*n* = 6), comparator not BMS (*n* = 4), and results were not presented at a disaggregated level (*n* = 3). In one case, we found that a full report [8] and a paper [9] reported results from the same analyses; data was therefore extracted from the full report. In another case, we found two papers with the same content and results and considered them as one paper [10, 11].

The 16 eligible studies were divided into five groups based on the type of DES that was evaluated and accounted for 498 separate analyses (Table 1). Four studies calculated the incremental cost-effectiveness ratio (ICER) for both PES and SES [2, 12–14], two studies [15, 16] focused on PES, three studies focused only on SES [17–19], and one study used ZES as the intervention [20]. The remaining six publications [8, 10, 11, 21–24] did not specifically identify the type of eluting drug under evaluation and calculated an ICER for a DES in general,

### Descriptive characteristics

**Fig. 2:**
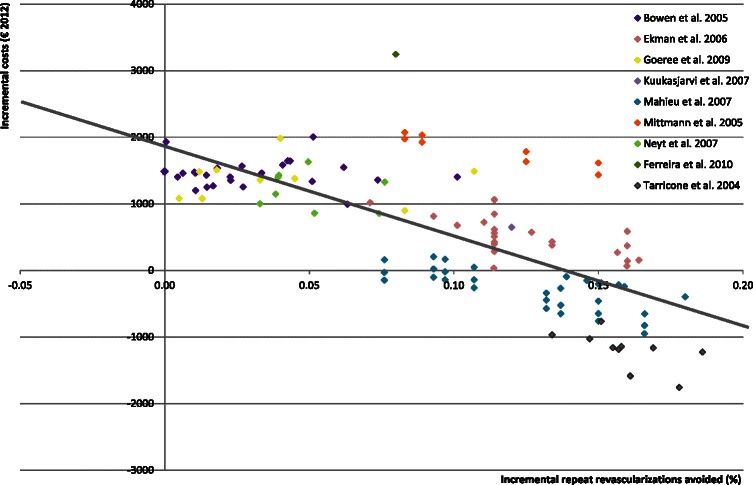
Cost-effectiveness plane, repeat revascularizations avoided

**Fig. 3:**
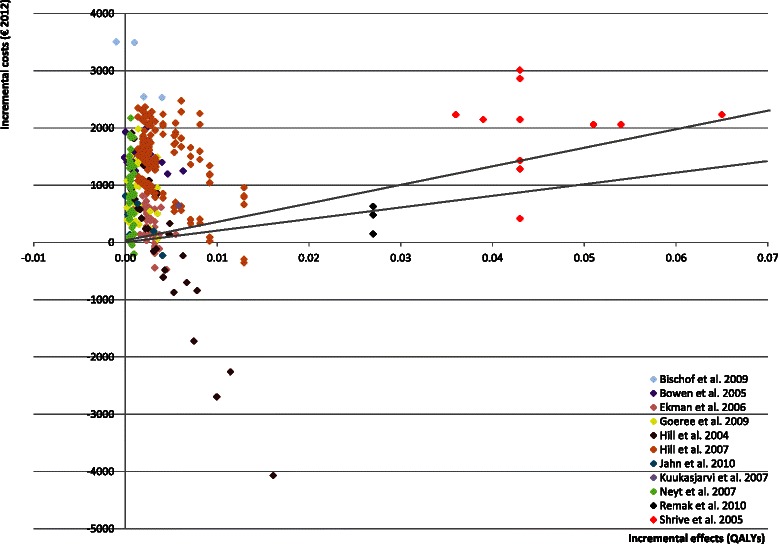
Cost-effectiveness plane, quality-adjusted life years gained. * The lines present the willingness to pay thresholds of 20,000 per QALY gained and 30,000 per QALY gained. The threshold in the Netherlands is between 20,000 - 80,000 per QALY gained [32]

In most analyses, DES was more expensive (88 % of analyses) and more effective in both QALYs and repeat revascularizations avoided (99 % of analyses) than BMS. Most of the 16 studies [2, 8, 10, 11, 14, 16, 21, 23] concluded that DES is not cost-effective for all subgroups since the incremental QALYs did not offset the incremental costs. However, many concluded that DES was more cost-effective in high-risk patients. The ICER varied considerably between and within studies: from DES being dominated by BMS [14, 21] to DES being dominant in specific analyses [2, 8, 10, 11, 15, 19, 22]. Figs. 2 and 3 present the variability of the incremental costs and effects of the studies using repeat revascularizations avoided or QALYs as an outcome measure, respectively. The mean values of input parameters stratified by the type of study outcome are presented in Table 2.

**Table 2:** Averages economic evaluations (univariate analyses)

	Total (CEAs & CUAs) (*N* = 16)	CEAs (*N* = 9)	CUAs (*N* = 11)
	Average ± SD	Average ± SD	Average ± SD
*Incremental outcomes*			
Incremental costs	€982 ± €894		
Incremental QALYs	0.0042 ± 0.008		
Incremental repeat revascularization avoided	0.0958 ± 0.0521		
*Input parameters*			
Number of stents per procedure	1.503 ± 0.367	1.382 ± 0.355	1.540 ± 0.364
Price of DES stent	€ 1,654 ± € 390	€ 1,912 ± € 672	€ 1,614 ± € 307
Price of BMS stent	€ 555 ± € 166	€ 670 ± € 307	€ 534 ± € 114
Price difference between stents	€ 1,085 ± € 337	€ 1,189 ± € 336	€ 1,056 ± € 331
Price of DES procedure (incl. stents)	€ 6,328 ± € 2,509	€ 7,811 ± € 1,475	€ 5,998 ± € 2,573
Price of BMS procedure (incl. stents)	€ 4,442 ± € 2,195	€ 6,259 ± € 1,536	€ 4,160 ± € 2,138
Cost difference between the procedures	€ 1,787 ± € 686	€ 1,551 ± € 805	€ 1,840 ± € 647
Probability restenosis BMS	0.142 ± 0.076	0.148 ± 0.055	0.140 ± 0.081
Probability restenosis DES	0.064 ± 0.038	0.056 ± 0.027	0.068 ± 0.041
Relative risk reduction DES vs. BMS	0.484 ± 0.204	0.578 ± 0.214	0.449 ± 0.189
Quality (0-100 %)*			
Total	59.5 ± 15.4		
Structure	62.5 ± 16.1		
Data	56.7 ± 21.6		
Consistency	55.1 ± 20.8		

* *N* = 16 studies

*CEA* cost-effectiveness analysis, *CUA* cost-utility analysis

We also assessed the quality of the models of all studies using the Philips et al. [7] checklist. Studies appeared to score higher on the theme structure (63 % ± 16 %) than on the other two themes, data (57 % ± 22 %) and consistency (55 % ± 21 %). The average overall quality of the models was moderate (59 % ± 15 % of a maximum possible score of 100 %).

### Outcome repeat revascularizations avoided

Based on 124 separate analyses (9 studies), the number of repeat revascularizations avoided (the absolute risk reduction in repeat revascularizations) with DES also varied considerably (Fig. 2) between and within studies (range: −0.0001, 0.19), which resulted in variation in the ICERs. The overall conclusions of most of the studies corresponded with the 124 separate analyses (Table 3). The regression analyses showed that the relative risk reduction of repeat revascularizations and the initial probabilities of restenosis were positively associated with repeat revascularizations avoided. Furthermore, a more complex vessel or lesion was associated with higher relative risk reduction and initial risk of restenosis after a percutaneous coronary intervention with BMS. Consequently, this leads to an increase in repeat revascularizations avoided and DES becomes more effective. Furthermore, the number of stents was also positively and significantly associated with repeat revascularizations avoided, probably because it is a proxy for subgroups who have a higher risk of developing restenosis due to diabetes, lesions and vessels characteristics. These factors could have been predicted beforehand since subgroup analyses and sensitivity analyses of the individual studies show the same conclusions.

**Table 3:** Associations between incremental revascularizations and covariates – DES vs BMS^a^

	Bivariate		
	∆ Repeat revascularization ^d^		
Covariates	β	N	se
		120	
*Population*			
Age		70	
Age >75	NA	0	NA
Age 65-75	−0.018	8	0.05
Age < 65	ref	62	
Complex lesion (yes vs. no)	0.029*	56	0.007
Complex vessel (yes vs. no)	0.042*	27	0.012
Multi vessel disease (yes vs. no)	0.019*	12	0.007
Diabetes (yes vs. no)	0.02*	64	0.007
Post MI (yes vs. no)	0.007	25	0.011
Elective (yes vs. no)	NA	0	NA
High risk (yes vs. no)	NA	0	NA
*Intervention*			
Type DES		120	
Sirolimus eluting stent	0.102*	21	0.014
Paclitaxel eluting stent	0.063*	56	0.014
Zotarolimus eluting stent	NA	0	NA
Drug eluting stent in general	ref	43	
*Study characteristics*			
Country		120	
Canada	−0.099	42	0.056
Sweden	−0.036	27	0.068
Brazil	−0.08	5	0.072
Finland	−0.04	1	0.072
Belgium	−0.07	39	0.059
Italy	ref	10	
Study year	0.01	120	0.008
Horizon >1 year (yes vs. no)	−0.006	120	0.021
Horizon (months) ^b^	<0.001		
Type of study (CUA vs. CEA)	NA	NA	NA
Model		120	
Markov model	NA	0	NA
Discrete event simulation model	NA	0	NA
Decision tree	NA	120	NA
Perspective		120	
Health care provider perspective	0.004	6	0.017
Health care sector perspective	0.04	31	0.05
Non-public perspective	NA	0	NA
Health care payer perspective	ref	83	
Funding		73	
No	0.034	27	0.045
Yes		46	
Both Industry and No industry	NA	0	NA
Industry	0.102*	37	0.046
No industry	ref	9	
Discounting (yes vs. no)^c^	−0.084*	11	0.026
*Input parameters*			
Number of stents used during the procedure	0.033*	111	0.01
Price difference between stents	NA	NA	NA
Price of BMS stent	NA	NA	NA
Price of DES stent	NA	NA	NA
Costs of BMS procedure (incl. stents)	NA	NA	NA
Costs of DES procedure (incl. stents)	NA	NA	NA
Difference in procedure costs	NA	NA	NA
**Probability of restenosis BMS**	0.521*	112	0.041
Probability of restenosis DES	0.436*	112	0.127
**Relative risk reduction repeat revascularization**	0.132*	112	0.018
Disutility of undergoing a CABG	NA	NA	NA
Disutility of undergoing a PCI	NA	NA	NA
Disutility of experiencing a MI	NA	NA	NA
Disutility for a patient with angina symptoms	NA	NA	NA
Quality of life of a patient with angina symptoms	NA	NA	NA
Quality of life of a patient after revascularization (recovered)	NA	NA	NA
Quality of life of a patient suffering from restenosis	NA	NA	NA
*Assumptions*			
Difference in clopidogrel (medication) usage (yes vs. no)	0.001	45	0.015
Wait time for revascularization included (yes vs. no)	−0.051	77	0.048
Repeat revascularization is based on angiographic follow-up data (yes vs. no)	0.082*	82	0.01
DES and BMS are not mixed up during a procedure	−0.061	120	0.047
Repeat interventions that occur during time horizon are the result of restenosis	NA	120	NA
There do not exist differences in mortality, thrombosis or MI between DES and BMS	0.039	120	0.039
The type of repeat revascularization is the same for the DES and BMS treatment groups	−0.071	120	0.044
There does not exist a difference in survival between DES and BMS	0.015	120	0.033
There does not exist a difference in thrombosis between DES and BMS	0.039	120	0.039
There does not exist a difference in MI between DES and BMS	0.046	120	0.031
*Quality of studies (Philips* et al. *2006)* [7]			
Structure (%)	−0.145	120	0.099
Data (%)	−0.167*	120	0.066
Consistency (%)	−0.153	120	0.081
Total (%)	−0.250*	120	0.087

^a^ Corrected for study; ^b^Shrive et al. & Remak et al. [17, 20] not included (lifetime horizon); ^c^ only studies with a time horizon longer than 1 year included; ^d^incremental repeat revascularization avoided; *p value < 0.05

*CEA* cost effectiveness analysis, *CUA* cost utility analysis, *DES* drug eluting stent, *MI* myocardial infarction, *NA* not applicable, *BMS* bare metal stent, *CABG* coronary artery bypass graft, *DES* drug eluting stent, *MI* myocardial infarction, *NA* not applicable, *PCI* percutaneous coronary intervention

Besides these factors that could be predicted beforehand, with the meta-regression analyses we were able to find a negative association between overall quality of a model and repeat revascularizations avoided. Furthermore, the theme data was also negatively associated with this incremental outcome. Consequently, models with a higher quality led to less favourable results for DES.

### Outcome of incremental QALYs

Figure 3 presents the incremental QALYs and incremental costs for 384 separate cost-effectiveness analyses (11 studies). This Figure shows that Shrive et al. [17] and Remak et al. [20] clearly found a larger incremental QALY gain than the other studies.

Again, the meta-regression analyses found associations with incremental QALYs that were expected (Table 4). Relative risk reduction of repeat revascularizations and the initial probability of restenosis after BMS were associated with a greater QALY gain, as seen in individual sensitivity analyses [2, 14, 15, 21, 22, 24]. Furthermore, analyses showed that non-elective patients, patients with a high risk of a repeat revascularization, patients with complex vessels or lesions or older patients will benefit more from DES, something that was also recognised in the individual studies [2, 12, 17, 21, 24]. In addition, we found a significant positive association between time horizon (continuous) and incremental QALYs. This was also found by Hill et al. [22] and Ekman et al. [15] who varied the time horizon in the sensitivity analyses.

**Table 4:** Associations between incremental QALYs and covariates – DES vs BMS^a^

	Bivariate		
	∆ QALYs		
Covariates	β	N	se
		384	
*Population*			
Age		190	
Age >75	0.029*	1	0.002
Age 65-75	0.015*	52	0.002
Age < 65	ref	137	
Complex lesion (yes vs. no)	0.001*	123	<0.001
Complex vessel (yes vs. no)	0.001*	51	<0.001
Multi vessel disease (yes vs. no)	0.001	90	<0.001
Diabetes (yes vs. no)	<0.001	135	<0.001
Post MI (yes vs. no)	<0.001	25	0.001
Elective (yes vs. no)	−0.001*	208	<0.001
High risk (yes vs. no)	0.004*	127	0.001
*Intervention*			
Type DES		384	
Sirolimus eluting stent	0.01	75	0.009
Paclitaxel eluting stent	0.011	151	0.009
Zotarolimus eluting stent	0.025	3	0.015
Drug eluting stent in general	ref	155	
*Study characteristics*			
Country		384	
United Kingdom	0.011	211	0.015
United States	0.001	4	0.019
Canada	0.016	72	0.015
Sweden	0.002	39	0.019
Austria	0.001	6	0.019
Finland	0.005	1	0.019
Belgium		51	
Study year	0.001	384	0.002
Horizon >1 year (yes vs. no)	0.002	384	0.001
Horizon (months) ^b^	<0.001*	373	<0.001
Type of study (CUA vs. CEA)	NA	NA	NA
Model		384	
Markov model	0.014	226	0.008
Discrete event simulation model	0.001	6	0.014
Decision tree	ref	152	
Perspective		384	
Health care provider perspective	0.006	7	0.012
Health care sector perspective	NA	0	NA
Non-public perspective	NA	0	NA
Health care payer perspective	ref	377	
Funding		333	
No	−0.001	30	
Yes		303	
Both Industry and No industry	0.043*	11	0.008
Industry	0.012	42	0.006
No industry	ref	250	
Discounting (yes vs. no)^c^	0.015	90	0.013
*Input parameters*			
Number of stents used during the procedure	0.001	379	0
Price difference between stents	NA	NA	NA
Price of BMS stent	NA	NA	NA
Price of DES stent	NA	NA	NA
Costs of BMS procedure (incl. stents)	NA	NA	NA
Costs of DES procedure (incl. stents)	NA	NA	NA
Difference in procedure costs	NA	NA	NA
Probability of restenosis BMS	0.024*	366	0.001
Probability of restenosis DES	0.005	282	0.004
Relative risk reduction repeat revascularization	0.007*	300	0.001
Disutility of undergoing a CABG	−0.747*	254	0.163
Disutility of undergoing a PCI	−0.107	254	0.433
Disutility of experiencing a MI	−0.021	40	0.097
Disutility for a patient with angina symptoms	−0.012	78	0.013
Quality of life of a patient with angina symptoms	−0.231*	338	0.04
Quality of life of a patient after revascularization (recovered)	−0.24*	380	0.024
Quality of life of a patient suffering from restenosis	−0.254*	144	0.031
*Assumptions*			
Difference in clopidogrel (medication) usage (yes vs. no)	<0.001	270	0.001
Wait time for revascularization included (yes vs. no)	−0.012*	336	0.006
Repeat revascularization is based on angiographic follow-up data (yes vs. no)	0.013*	329	0.006
DES and BMS are not mixed up during a procedure	0.002	384	0.01
Repeat interventions that occur during time horizon are the result of restenosis	0.02*	384	0.01
There do not exist differences in mortality, thrombosis or MI between DES and BMS	−0.003	384	0.016
The type of repeat revascularization is the same for the DES and BMS treatment groups	−0.008	384	0.016
There does not exist a difference in survival between DES and BMS	0.001	384	0.002
There does not exist a difference in thrombosis between DES and BMS	−0.003	384	0.016
There does not exist a difference in MI between DES and BMS	−0.006	384	0.01
*Quality of studies (Philips* et al. *2006)* [7]			
Structure (%)	−0.006	384	0.033
Data (%)	0.006	384	0.024
Consistency (%)	−0.018	384	0.02
Total (%)	<0.001	384	0.032

^a^ Corrected for study; ^b^Shrive et al. & Remak et al. [17, 20] not included (lifetime horizon); ^c^ only studies with a time horizon longer than 1 year included; * *p* value < 0.05

*CEA* cost effectiveness analysis, *CUA* cost utility analysis, *DES* drug eluting stent, *MI* myocardial infarction, *NA* not applicable, *BMS* bare metal stent, *CABG* coronary artery bypass graft, *DES* drug eluting stent, *MI* myocardial infarction, *NA* not applicable, *PCI* percutaneous coronary intervention

Studies [2, 17] that have explicitly mentioned that they have assumed that the occurrence of repeat revascularizations within the time horizon is the result of restenosis and studies assuming that repeat revascularization rates are based on angiographic follow-up have estimated significantly higher incremental QALYs. Angiographic follow-up leads to inflated estimates of clinical effectiveness compared with clinical follow-up since not clinically significant restenosis results in “unnecessary” repeat revascularizations when angiographic follow-up is performed. Consequently, the difference in repeat revascularizations will be overestimated (oculo-stenotic effect) [25]. Some studies use “real-world” [8, 10, 11, 21] follow-up data and consequently report lower estimates (visible in Figs. 2 and 3) than other studies such as, Remak et al. [20] that used angiographic follow-up [12, 15, 17, 23]. This phenomenon is described earlier by Eisenberg et al. [26], who concluded that cost-effectiveness studies using angiographic follow-up overestimate the cost-effectiveness of DES.

The meta-regression analyses showed that studies using real-world evidence compared with angiographic follow-up leads to a reduction in incremental QALY gain. The added value of meta-regression analyses is limited in explaining the variation in incremental QALYs, although it identified modelling assumptions that were significantly associated with incremental QALYs.

### Outcome incremental costs

Figures 2 and 3 show that there was large variation in incremental costs (range: €-4070 to €3506). Regression analyses (Table 5) confirmed associations (cost parameters and population characteristics) that were seen in the individual studies [2, 8, 12, 17, 20, 21, 24]. The analyses showed that probability of restenosis after BMS, the reduction in restenosis risk by DES, the difference in stent price, and the number of stents used were important parameters influencing the incremental costs. Both input parameters varied considerably between the analyses: the difference in stent costs ranged from €0 [8, 19] to €2685 [17] and the number of stents varied between 1 [22] and 2.6 [19] stents per procedure depending on the type of patient.

**Table 5:** Associations between incremental costs and covariates – DES vs BMS^a^

	Bivariate		
	∆ Costs (2012€)		
Covariates	β	N	se
		437	
*Population*			
Age		190	
Age >75	315	1	901
Age 65-75	−31	52	695
Age < 65	ref	137	
Complex lesion (yes vs. no)	172*	134	85
Complex vessel (yes vs. no)	−5	62	116
Multi vessel disease (yes vs. no)	122	98	200
Diabetes (yes vs. no)	−217*	150	78
Post MI (yes vs. no)	−88	25	88
Elective (yes vs. no)	346*	208	109
High risk (yes vs. no)	−291	127	193
*Intervention*			
Type DES		437	
Sirolimus eluting stent	551	100	636
Paclitaxel eluting stent	379	180	636
Zotarolimus eluting stent	−324	3	1321
Drug eluting stent in general	ref	154	
*Study characteristics*			
Country		437	
United Kingdom	2147*	211	836
United States	4425*	4	1050
Canada	2922*	79	808
Sweden	1745	39	1016
Brazil	3444*	5	932
Austria	1752	6	1035
Finland	2051	1	1174
Belgium	1698	82	879
Italy	ref	10	
Study year	−190	437	137
Horizon >1 year (yes vs. no)	−479	437	277
Horizon (months) ^b^	−32*	414	6
Type of study (CUA vs. CEA)	−194*	507	86
Model		437	
Markov model	613	230	611
Discrete event simulation model	−435	6	1219
Decision tree	ref	201	
Perspective		437	
Health care provider perspective	266	14	363
Health care sector perspective	−1332	31	1151
Non-public perspective	−1057	2	670
Health care payer perspective	ref	390	
Funding		347	
No	1480*	31	634
Yes		316	
Both Industry and No industry	1246	11	1041
Industry	−621	56	663
No industry	ref	249	
Discounting (yes vs. no)^c^	1071	91	713
*Input parameters*			
Number of stents used during the procedure	708*	424	83
Price difference between stents	1.264*	418	0.13
Price of BMS stent	0.503*	320	0.354
Price of DES stent	1.001*	312	0.152
Costs of BMS procedure (incl. stents)	0.339*	278	0.092
Costs of DES procedure (incl. stents)	0.412*	278	0.053
Difference in procedure costs	0.799*	278	0.075
Probability of restenosis BMS	−3072*	407	322
Probability of restenosis DES	−1907*	323	899
Relative risk reduction repeat revascularization	−1676*	341	250
Disutility of undergoing a CABG	NA	NA	NA
Disutility of undergoing a PCI	NA	NA	NA
Disutility of experiencing a MI	NA	NA	NA
Disutility for a patient with angina symptoms	NA	NA	NA
Quality of life of a patient with angina symptoms	NA	NA	NA
Quality of life of a patient after revascularization (recovered)	NA	NA	NA
Quality of life of a patient suffering from restenosis	NA	NA	NA
*Assumptions*			
Difference in clopidogrel (medication) usage (yes vs. no)	181	279	216
Wait time for revascularization included (yes vs. no)	−733	347	486
Repeat revascularization is based on angiographic follow-up data (yes vs. no)	−593	372	492
DES and BMS are not mixed up during a procedure	−542	437	741
Repeat interventions that occur during time horizon are the result of restenosis	855	437	841
There do not exist differences in mortality, thrombosis or MI between DES and BMS	−980	437	878
The type of repeat revascularization is the same for the DES and BMS treatment groups	501	437	1187
There does not exist a difference in survival between DES and BMS	−238	437	426
There does not exist a difference in thrombosis between DES and BMS	−589	437	754
There does not exist a difference in MI between DES and BMS	−595	437	665
*Quality of studies (Philips* et al. *2006)* [7]			
Structure (%)	2154	437	1819
Data (%)	1670	437	1318
Consistency (%)	718	437	1463
Total (%)	2761	437	1804

^a^ Corrected for study; ^b^Shrive et al. & Remak et al. [17, 20] not included (lifetime horizon); ^c^ only studies with a time horizon longer than 1 year included; * *p* value < 0.05

*CEA* cost effectiveness analysis, *CUA* cost utility analysis, *DES* drug eluting stent, *MI* myocardial infarction, *NA* not applicable, *BMS* bare metal stent, *CABG* coronary artery bypass graft, *DES* drug eluting stent, *MI* myocardial infarction, *NA* not applicable, *PCI* percutaneous coronary intervention

On a meta-level we were able to conclude that funding and the type of cost-effectiveness analysis was associated with incremental costs. Funding was provided by the stent manufacturer in five [15, 17–20] of the 16 studies and three of them [15, 17, 20] concluded that DES was cost-effective compared with BMS. Of the studies that were not *funded by a manufacturer* (*N* = 8) only one [10, 11] of them concluded that DES could be cost-effective. Studies that were not funded estimated on average higher incremental costs than studies that were (*p* < 0.05). Furthermore, some associations with incremental costs are recognised from scenario analyses performed by studies. The directions of the following associations are confirmed by the regression analysis but not significant. According to Jahn et al. [10, 11] it is important to incorporate wait time into the model since it leads to a decrease (−734, 95 % CI:-1690;223) in incremental costs. A time horizon shorter than 12 months was associated with higher incremental costs (479, 95 % CI: −1024;65); Hill et al. [22] estimated costs and effects for different time horizons and showed that a longer time horizon led to lower incremental costs. This is likely because of the continuing treatment effect of DES in the subsequent years which would increase in the number of repeat revascularizations avoided compared with BMS. This increase in reduction of repeat revascularization would further offset the cost of the initially more expensive DES.

Meta-regression analyses showed also that the number of repeated revascularizations avoided explained a large proportion of variation (R^2^ = 0.53). As shown in Fig. 2, there appeared to be a linear association between incremental costs and repeat revascularizations avoided. Incremental QALYs (Fig. 3), on the other hand, was not associated with incremental costs (R^2^ = 0.001), probably since incremental QALYs are determined by several factors including repeat revascularizations avoided, life-years gained and quality of life values.

## Discussion

This study explored the usefulness of meta-regression analyses in combination with a systematic review of economic evaluations compared with conventional review methods. The aim of conventional systematic reviews is to show relevant publications on the cost-effectiveness of certain treatments in a systematic manner. When possible, conventional reviews describe associations between study characteristics, input parameters and outcomes. However, it is not possible to statistically determine if the association actually exists, which covariates explains the variation best, to correct for interactions or to predict the incremental outcomes. This case study was inspired by meta-analyses of treatment effectiveness studies that are frequently performed to obtain a single summary estimate. More interesting than meta-analyses are meta-regression analyses that try to relate the size of treatment effect to one or more characteristics of the included studies [1]. Using meta-regression analyses to explore the associations between incremental outcomes and input parameters is unique for a systematic review of economic evaluations and could help to explain variation in cost-effectiveness outcomes between studies. We used meta-regression analyses to explain the variability in the outcomes of cost-effectiveness studies (i.e., incremental costs and effects) of DES versus BMS and found that, besides confirming associations that could be predicted from individual studies, associations at a meta-level also exist, such as an association between outcomes and the quality of the models.

The most important factors that were associated with the results were patient characteristics (age, vessel, lesion), procedure (type of stent and elective versus non-elective), specific input parameters (number of stents per procedure, cost per stent/procedure, restenosis risk with BMS and the efficacy of DES) and the quality of the models. Many of these associations had already been reported in the studies themselves, which can be seen as evidence that the meta-regression produced valid results. However, besides these previously reported associations, we also found associations between study outcomes and the quality of the model, time horizon, efficacy assumptions, and funding which could only be identified at a ‘meta level’. Moreover, this review identified an association between the incremental costs and absolute risk reduction in repeat revascularizations on ‘meta-level’ (Fig. 2) showing the added value of meta-regression analyses.

Some of the associations we found are desirable since they involve parameters that influence the results and that can be controlled by clinicians and policymakers. For example, factors like the costs of a stent are expected to be associated with the results. Other factors such as patient characteristics can be changed by means of patient selection. However, the presence of other associations such as the quality of the models, assumptions, time horizon or funding raises concerns. Moreover, other parameters were not significantly associated with outcomes (e.g., wait time and incremental costs, or funding and incremental QALYs). These parameters could have influenced the outcomes but are undesirable since e.g., funding should not play a role in the outcomes of the study. It is important for authors to follow the recommendations of the ISPOR-SMDM task force for modelling good research practices [27] and the recommendations based on the Philips et al. checklist [7] for modelling studies to increase the quality of the study and generalizability of the results.

### Limitations

Despite the fact that the quality of the models was assessed by two independent reviewers it was difficult to judge the quality due to subjectivity of the questions; this problem was been recognized in the past [28]. Furthermore, to provide studies with a score between 0 and 100 % we needed to assume that all questions of the checklist were equally important. Thus studies could obtain a reasonably high score if less informative/important questions were fulfilled. In addition, the quality of the models was based on the documentation of the model and therefore it is possible that studies that scored low did not transparently present model details, however the actual model could be of high quality. Regardless of these limitations, we found a statistically significant association between quality and the outcome repeat revascularization.

Furthermore, title abstract screening was performed by one reviewer which could be seen as a limitation of the study. However, checks of whether the studies included in previously published reviews were also identified with the search, increased the sensitivity of the search and thereby reduced the chance of missing relevant publications. Full assessment and assessing the quality of the model using the Philips checklist was performed by two reviewers independently.

Another limitation of our study is that all 508 analyses were analysed as independent observations even though in reality these 508 analyses were based on 16 studies. We have used study identification number as a random effect in the regression models to address this problem.

In this case study, linear regression models were used to estimate the associations of study characteristics on the outcomes (incremental costs, incremental QALYs and repeat revascularizations avoided) since the number of observations was large. However, regression models could be improved by first considering if the dependent variable (e.g., incremental costs) can best be modelled using a different function (e.g., gamma).

Moreover, meta-regression analyses (bivariate or multivariate) help to explain variation in outcomes, however it also identified associations that were not expected a priori. For example, type of study was associated with the incremental costs, which is not logical since the type of study mainly influences the incremental effects. Covariates that are on beforehand implausible (e.g., type of study and incremental costs) should not be included in future meta-regression analyses since it leads to false positive outcomes.

In addition, transparency in documentation is a major issue leading to a high frequency of missing values that made it impossible to perform multivariate analyses with all of the parameters that were significant in the bivariate analyses. Consequently, we were unable to: 1) take into account interaction effects, 2) determine the most influential covariates, and 3) create a prediction model. A solution could be to include a smaller number of input parameters with only common input parameters (e.g., cost of procedure, time horizon etc.). However, this will lead to fewer associations between outcomes and covariates.

Transparent reporting is crucial in this field and would solve the problem of missing values for systematic reviews such as this. A recently published review on the challenges of modelling the cost-effectiveness of cardiovascular disease interventions has recognized the same problem [29].

Lastly, we did not include the studies published after January 2012. However, we expect that including newer studies that met inclusion criteria (i.e., estimating the cost-effectiveness of DES versus BMS using modelling methods) do not have an impact on the results of our case study showing that using meta-regression analyses could be useful method in addition to conventional systematic reviews.

To improve this case study lessons can be learned from meta-regression analyses and meta-analyses that are performed for the clinical effectiveness of interventions. More specifically, it could provide guidance in how to handle missing data [30], how to treat study heterogeneity, how to include covariate interaction [31]. In addition, it shows limitations of the methods [1].

## Conclusions

This study has showed that meta-regression analyses can be used to explore relationships between study characteristics and cost-effectiveness outcomes and can draw from the methodology used in other fields even though it is not yet fully developed. Compared with conventional review methods or sensitivity analyses of individual studies meta-regression analyses can be of added value since it identifies significant associations that could not be identified before. The quality of the models was associated with the outcomes of the studies and therefore it is important that a quality check is performed before interpreting the results of the study.

## Additional files

Additional file 1: **Search string.** (DOCX 15 kb)

Additional file 2: **PRISMA guidelines.** (PDF 514 kb)

## Abbreviations

BMS: Bare-metal stents
CEA: Cost-effectiveness analysis
DES: Drug-eluting stents
ICER: Incremental cost-effectiveness ratio
PES: Paclitaxel-eluting stent
QALY: Quality-adjusted life years
SES: Sirolimus-eluting stent
TLR: Target lesion revascularization
TVR: Target vessel revascularization
ZES: Zotarolimus-eluting stent

## References

[1] Thompson SG, Higgins JP (2002). How should meta-regression analyses be undertaken and interpreted?. Stat Med.

[2] Hill RA, Boland A, Dickson R, Dundar Y, Haycox A, McLeod C (2007). Drug-eluting stents: a systematic review and economic evaluation. Health Technol Assess.

[3] Neyt M, Van Brabandt H, Devriese S, De Laet C (2009). Cost-effectiveness analyses of drug eluting stents versus bare metal stents: A systematic review of the literature. Health Policy.

[4] Ligthart S, Vlemmix F, Dendukuri N, Brophy JM (2007). The cost-effectiveness of drug-eluting stents: A systematic review. Can Med Assoc J.

[5] Purchasing Power Parities and exchange rate for OECD countries. 2011; Available at: http://stats.oecd.org/Index.aspx?DataSetCode=SNA_TABLE4. Accessed January 15, 2011.

[6] Statistics Netherlands. Consumer price index. 2012; Available at: http://statline.cbs.nl/StatWeb/publication/?VW=T&DM=SLNL&PA=71311ned&D1=a&D2=0&D3=12,25,38,51,64,77,90,103,116,129,142,155,168,181-220&HD=120217-1502&HDR=G1,T&STB=G2, 2012.

[7] Philips Z, Bojke L, Sculpher M, Claxton K, Golder S (2006). Good practice guidelines for decision-analytic modelling in health technology assessment: a review and consolidation of quality assessment. Pharmacoeconomics.

[8] Neyt M, Van Brabandt H, Devriese S, Mahieu J, De Ridder A, De Graeve D, et al. Drug Eluting Stents in Belgium: Health Technology Assessment. Health Technology Assessment (HTA). Bruxelles: Belgian Health Care Knowledge Centre (KCE); 2007. KCE reports 66C (D/2007/10.273/49). https://kce.fgov.be/sites/default/files/page_documents/d20071027349.pdf.

[9] Neyt M, De Laet C, De Ridder A, Van Brabandt H (2009). Cost Effectiveness of Drug-Eluting Stents In Belgian Practice Healthcare Payer Perspective. Pharmacoeconomics.

[10] Jahn B, Pfeiffer KP, Theurl E, Tarride JE, Goeree R (2010). Capacity Constraints and Cost-Effectiveness: A Discrete Event Simulation for Drug-Eluting Stents. Med Decis Mak.

[11] Jahn B, Theurl E, Siebert U, Pfeiffer KP (2010). Tutorial in medical decision modeling incorporating waiting lines and queues using discrete event simulation. Value Health.

[12] Mahieu J, de Ridder A, de Graeve D, Vrints C, Bosmans J (2007). Economic analysis of the use of drug-eluting stents from the perspective of Belgian health care. Acta Cardiol.

[13] Mittmann N, Brown A, Seung SJ, Coyle D, Cohen E, Brophy J, et al. Economic evaluation of drug eluting stents [Technology report no 53]. Ottawa: Canadian Coordinating Office for Health Technology Assessment; 2005. https://www.cadth.ca/media/pdf/272_drug_eluting_stents_tr_e.pdf.

[14] Bischof M, Briel M, Bucher HC, Nordmann A (2009). Cost-Effectiveness of Drug-Eluting Stents in a US Medicare Setting: A Cost-Utility Analysis with 3-Year Clinical Follow-Up Data. Value Health.

[15] Ekman M, Sjogren I, James S (2006). Cost-effectiveness of the Taxus paclitaxel-eluting stent in the Swedish healthcare system. Scand Cardiovasc J.

[16] Ferreira E, Araujo DV, Azevedo VM, Rodrigues CV, Ferreira A, Junqueira Cde L (2010). Analysis of the cost-effectiveness of drug-eluting and bare-metal stents in coronary disease. Arq Bras Cardiol.

[17] Shrive FM, Manns BJ, Galbraith PD, Knudtson ML, Ghali WA (2005). Economic evaluation of sirolimus-eluting stents. CMAJ.

[18] Polanczyk CA, Wainstein MV, Ribeiro JP (2007). Cost-effectiveness of sirolimus-eluting stents in percutaneous coronary interventions in Brazil. Arq Bras Cardiol.

[19] Tarricone RR (2004). What reimbursement for coronary revascularization with drug-eluting stents?. Eur J Health Econ.

[20] Remak E, Manson S, Hutton J, Brasseur P, Olivier E, Gershlick A (2010). Cost-effectiveness of the Endeavor stent in de novo native coronary artery lesions updated with contemporary data. Euro Intervention.

[21] Bowen J, Hopkins R, He Y, Blackhouse G, Lazzam C, Tu J, et al. Systematic review and cost-effectiveness analysis of drug eluting stents compared to bare metal stents for percutaneous coronary interventions in Ontario. Interim Report for the Ontario Ministry of Health and Long-term Care. Hamilton, ON: Program for Assessment of Technology in Health, McMaster University; 2005. http://www.path-hta.ca/Libraries/Reports/DESreport.sflb.ashx

[22] Hill R, Bagust A, Bakhai A, Dickson R, Dundar Y, Haycox A. Coronary artery stents: a rapid systematic review and economic evaluation. Health Technol Assess. 2004;8(35):1–256. 10.3310/hta8350 15361315

[23] Kuukasjarvi P, Rasanen P, Malmivaara A, Aronen P, Sintonen H (2007). Economic evaluation of drug-eluting stents: A systematic literature review and model-based cost-utility analysis. Int J Technol Assess Health Care.

[24] Goeree R, Bowen JM, Blackhouse G, Lazzam C, Cohen E, Chiu M (2009). Economic evaluation of drug-eluting stents compared to bare metal stents using a large prospective study in Ontario. Int J Technol Assess Health Care.

[25] Ruygrok PN, Melkert R, Morel MAM, Ormiston JA, Bar FW, Fernandez-Aviles F (1999). Does angiography six months after coronary intervention influence management and outcome?. J Am Coll Cardiol.

[26] Eisenberg MJ (2006). Drug-eluting stents - The price is not right. Circulation.

[27] Caro JJ, Briggs AH, Siebert U, Kuntz KM (2012). ISPOR-SMDM Modeling Good Research Practices Task Force. Modeling good research practices--overview: a report of the ISPOR-SMDM Modeling Good Research Practices Task Force--1. Value Health.

[28] Handels RL, Wolfs CA, Aalten P, Joore MA, Verhey FR, Severens JL. Diagnosing Alzheimer’s disease: A systematic review of economic evaluations. Alzheimers Dement. 2014;10(2):225-37. 10.1016/j.jalz.2013.02.005 23727080

[29] Burgers LT, Redekop WK, Severens JL. Challenges in Modelling the Cost Effectiveness of Various Interventions for Cardiovascular Disease. Pharmacoeconomics. 2014;32(7):627-37. 10.1007/s40273-014-0155-9 24748448

[30] Higgins JPT, White IR, Wood AM. Imputation methods for missing outcome data in meta-analysis of clinical trials. Clin Trials. 2008;5(3):225–39. doi:10.1177/1740774508091600. 10.1177/1740774508091600 PMC2602608 18559412

[31] Donegan S, Williams L, Dias S, Tudur-Smith C, Welton N. Exploring treatment by covariate interactions using subgroup analysis and meta-regression in cochrane reviews: a review of recent practice. PLoS One. 2015;10(6):e0128804. 10.1371/journal.pone.0128804 PMC4452239 26029923

[32] Council for Public Health and Health Care (RVZ). Fair and sustainable care. 2006. http://www.raadrvs.nl/uploads/docs/Advies_-_Zinnige_en_duurzame_zorg.pdf.

