# Supplementary material for: Using meta-regression analyses in addition to conventional systematic review methods to examine the variation in cost-effectiveness results – a case study
Source: BMC Health Serv Res. 2016 Jan 20;16:23. doi: 10.1186/s12913-015-1230-4 (PMC4719667; doi:10.1186/s12913-015-1230-4)
Supplement: Supplementary file 1 — Search string. (DOCX 15 kb) [file 12913_2015_1230_MOESM1_ESM.docx]

**Supplementary material**

**Appendix – search terms**

Pubmed

#1 cardiovascular disease[MeSH]

#2 stent*

#3 economics

#4 econom*

#5 cost

#6 costs

#7 costly

#8 costing

#9 price

#10 prices

#11 pricing

#12 pharmacoeconomics

#13 pharmacoecon*

#14 expenditure*

#15 energy

#16 #13 NOT #14

#17 "value for money"

#18 budget*

#19 #3 OR #4 OR #5 OR #6 OR #7 OR #8 OR #9 OR #10 OR #11 OR #12 OR #15 OR #16 or #17 OR #18

#20 Humans[Mesh]

#21 "1990"[PDat] : "2012"[PDat]

#22 English[lang]

#23 #1 AND #2 AND #19 AND #20 AND #21 AND #22

Embase

#1 'cardiovascular disease'/exp

#2 'stent'/exp

#3 'economics'/exp

#4 'cost'/exp

#5 costly

#6 costing

#7 price

#8 prices

#9 pricing

#10 pharmacoeconomics

#11 'pharmacoeconomics'/exp

#1 2 'value for money'

#13 'budget'/exp

#14 #3 OR #4 OR #5 OR #6 OR #7 OR #8 OR #9 OR #10 OR #11 OR #12 OR #13

#15 [humans]/lim

#16 [1-1-1990]/sd NOT [31-12-2011]/sd

#17 [english]/lim

#18 #1 AND #2 AND #14 AND #15 AND #16 AND #17

Cochrane (43) CRD (205) and INAHTA (24) are checked for relevant publications

Economic filter:

Centre for Reviews and Dissemination. NHS EED Economics Filter [Internet]. York: Centre for Reviews and Dissemination; 2010. Available from: http://www.york.ac.uk/inst/crd/intertasc/nhs_eed_strategies.html
